# *In*-class transition (*i*CT) of proteasome inhibitor-based therapy: a community approach to multiple myeloma management

**DOI:** 10.1038/s41408-023-00912-9

**Published:** 2023-09-19

**Authors:** Robert M. Rifkin, Saulius K. Girnius, Stephen J. Noga, Ruemu E. Birhiray, Suman Kambhampati, Sudhir Manda, Roger M. Lyons, Habte A. Yimer, Dasha Cherepanov, Eric Lloyd, Presley Whidden, Joshua Richter

**Affiliations:** 1 Rocky Mountain Cancer Centers/US Oncology Research, Denver, CO USA; 2 Trihealth Cancer Institute, Cincinnati, OH USA; 3 Takeda Pharmaceuticals U.S.A., Inc., Lexington, MA USA; 4 Hematology Oncology of Indiana/American Oncology Network, Indianapolis, IN USA; 5 Kansas City Veterans Affairs Medical Center, Kansas City, MO USA; 6 Arizona Oncology/US Oncology Research, Tucson, AZ USA; 7 Texas Oncology/US Oncology Research, San Antonio, TX USA; 8 Texas Oncology/US Oncology Research, Tyler, TX USA; 9 Takeda Development Center Americas, Inc. (TDCA), Lexington, MA USA; 10 Takeda Pharmaceuticals U.S.A., Inc., Bannockburn, IL USA; 11 Tisch Cancer Institute, Icahn School of Medicine at Mount Sinai, New York, NY USA

**Keywords:** Cancer therapy, Myeloma, Combination drug therapy, Phase IV trials

## Abstract

Long-term proteasome inhibitor (PI) treatment can improve multiple myeloma (MM) outcomes, but this can be difficult to achieve in clinical practice due to toxicity, comorbidities, and the burden of repeated parenteral administration. US MM-6 (NCT03173092) enrolled transplant-ineligible patients with newly diagnosed MM to receive all-oral ixazomib-lenalidomide-dexamethasone (IRd; ≤39 cycles or until progression or toxicity) following three cycles of bortezomib-based induction. Primary endpoint: 2-year progression-free survival (PFS). Key secondary/exploratory endpoints included overall response rate (ORR), overall survival (OS), safety, quality of life (QoL), treatment satisfaction, and actigraphy. At datacut, in the fully accrued cohort of 140 patients, median age was 73 years with 42% aged ≥75 and 61% deemed frail; 10% of patients were ongoing on treatment. After a median follow-up of 27 months, the 2-year PFS rate was 71% (95% confidence interval: 61–78). ORR increased from 62% at the end of induction to 80% following *in-*class transition (*i*CT) to IRd for a median of 11 months. The 2-year OS rate was 86%. The overall safety profile/actigraphy levels were consistent with previous reports; QoL/treatment satisfaction scores were stable with ongoing therapy. *i*CT to IRd may allow prolonged PI-based therapy with promising efficacy and a tolerable safety profile, while maintaining QoL.

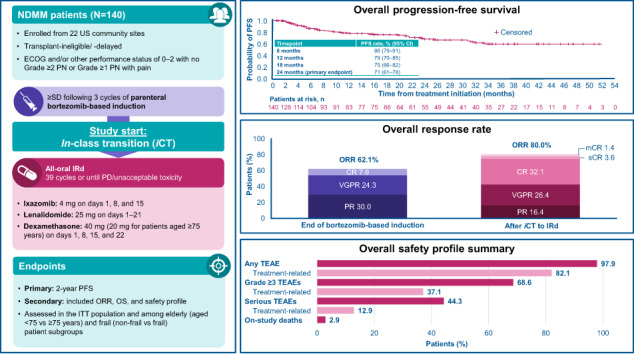

## Introduction

Long-term parenteral proteasome inhibitor (PI)-based regimens are a cornerstone of treatment among patients with multiple myeloma (MM) [1, 2]. For transplant-ineligible patients with newly diagnosed MM (NDMM), the addition of PIs to 2-drug standard of care treatment regimens has improved progression-free survival (PFS) and overall survival (OS) rates in phase 3 clinical studies [3–6]. However, prolonged therapy with parenteral PIs can be difficult to achieve in routine clinical practice due to unfavorable toxicity [7], existing comorbidities [8–10], the burden of repeated, clinic-based treatment administration, difficulty traveling to treatment centers, and patient preference for treatment outside of a clinic [11]. Such factors are likely to impact older and frail patients, who comprise a large proportion of all individuals with MM [12, 13]. By comparison, due to strict eligibility criteria, patient cohorts enrolled in randomized controlled trials (RCTs) are often younger and healthier versus real-world patient populations, and not always representative of the wider MM population [13].

Ixazomib is an oral PI, approved in combination with lenalidomide-dexamethasone (Rd) for the treatment of MM patients who have received ≥1 prior therapy [14, 15]. Ixazomib-lenalidomide-dexamethasone (IRd) has demonstrated prolonged PFS versus placebo-Rd and a tolerable safety profile in both relapsed/refractory MM (RRMM) and transplant-ineligible patients with NDMM [16, 17], although in the study of RRMM, no OS benefit was demonstrated at a median follow-up of 23 months, with median OS not reached in both the IRd and placebo treatment arms [16]. To facilitate continuous, long-term PI-based treatment and assess its potential benefit in a patient cohort representative of that found in routine clinical practice (including older, frail, and comorbid patients), the community-based US MM-6 study was designed to investigate *in*-class transition (*i*CT) from parenteral bortezomib-based induction to all-oral IRd therapy in patients with NDMM (NCT03173092) [18]. The objective is to increase duration of PI-based therapy and improve outcomes, while maintaining patient quality of life (QoL) and a tolerable safety profile. Here we report fully accrued data from US MM-6.

## Methods

### Study design and patient eligibility

Full methods have been previously published [18]. US MM-6 was designed as a prospective, open-label, single-arm, phase 4 study in a community-based population; at data accrual, enrollment was complete. Patients were enrolled at 22 US community sites, including three Veterans’ Affairs sites. Adults with NDMM who were ineligible for transplantation, or whose transplantation was delayed for ≥2 years, were eligible. Following three cycles of bortezomib-based induction, patients were administered oral IRd (planned dosages: ixazomib 4 mg on days 1, 8, and 15; lenalidomide 25 mg on days 1–21; and dexamethasone 20–40 mg on days 1, 8, 15, and 22) in 28-day cycles, for 39 cycles or until toxicity or progressive disease (PD). Patients were permitted to continue IRd beyond cycle 39 if investigators deemed a clinical benefit with further treatment. Following end-of-treatment assessment (performed within 30 days of the last ixazomib dose), patients entered a follow-up period to evaluate PFS and OS until PD or death, loss to follow-up, or study termination.

US MM-6 was conducted in accordance with the International Council on Harmonization Good Clinical Practice guidelines, the ethical principles that have their origins in the Declaration of Helsinki, and applicable regulatory requirements. Local or central institutional review boards for each study center approved the present study. All patients gave written informed consent to be included, and to the use of a wearable device to capture digital actigraphy data and a smartphone application for electronic patient reported outcomes (ePROs).

### Endpoints and assessments

The primary endpoint was 2-year PFS among the intent-to-treat (ITT) population, defined as the time from IRd initiation to the first documentation of PD, or death from any cause; PD was determined based on local laboratory test results and investigator assessed modified International Myeloma Working Group (IMWG) response criteria [19]. Secondary endpoints included ixazomib and IRd treatment durations, defined as the times from the first administration of ixazomib and IRd, respectively, to the final date of administration of ixazomib or any of the drugs in the IRd regimen, respectively; response rates, also based on investigator assessed modified IMWG criteria [19] (for complete response [CR], bone marrow plasma cell percentage was measured by aspiration and/or biopsy); OS, defined as the time from IRd initiation to death from any cause; safety, assessed by Medical Dictionary for Regulatory Activities (version 22.0) preferred terms and graded according to the National Cancer institute Common Terminology Criteria for adverse events (AEs; version 4.03); and ePRO data, specifically global health status (GHS) / QoL and treatment satisfaction, which were assessed via electronic questionnaires (European Organisation for Research and Treatment of Cancer quality of life questionnaire [EORTC QLQ-C30], derived from items 29 and 30; EORTC QoL questionnaire MM module [EORTC QLQ-MY20, item 43 on peripheral neuropathy]; and Treatment Satisfaction Questionnaire for Medication-9 [TSQM-9]) using a smartphone application. Exploratory endpoints included actigraphy analyses of patient sleep duration and activity based on duration and step counts, collected automatically via digital devices worn by patients (Garmin Vivofit 3 activity tracker). Details on ePRO scoring for US MM-6 have been reported previously [18].

### Statistical analysis

Planned enrollment was 160 patients, which provides 90% power with a one-sided α value of 0.05, to demonstrate a 2-year PFS rate of 62%, exceeding the 2-year PFS rate of 50% derived from historical controls [3, 20, 21]. Enrollment slowed during the COVID pandemic, leading to a decision to curtail enrollment at 140 patients.

Kaplan-Meier (KM) methodology was used to estimate the 2-year PFS rate, survival curves, and medians, alongside associated 95% confidence intervals (CIs). Median treatment durations were ‘simple’, not calculated using the KM method. For ePRO analysis, the mean change from the baseline (assessed at the end of the first cycle) over the treatment period was described among patients who had completed the initial questionnaire, as well as at ≥1 post-baseline date. To ensure analysis captured the same ePRO assessment periods across patients, only data captured between day 22 of each cycle, and day 3 of the following cycle were used for analysis to account for off-schedule launch of ePRO questionnaires that may have occurred at different sites. Actigraphy devices had to be worn for ≥14 days per treatment cycle for data collection to be compliant; compliant days were not necessarily consecutive. For each compliant day, the device had to be worn for ≥12 hours (i.e., the patient had been recorded as moving for ≥12 hours that day, assuming that even during sleep or rest, a low level of movement would have been recorded). Days for which actigraphy data were available, but during which the patient had not been recorded moving for ≥12 hours, were excluded from analyses; this allowed the exclusion of data on days the devices were likely not being worn, and hence avoided wrongful classification of these patients as ‘sedentary’. For actigraphy analysis, outlying datapoints (defined as >4 standard deviations [StDevs] from the mean) were removed; recalculation of the mean and StDev was then performed until there were no outliers. For all endpoints, subgroup analyses of outcomes by patient age (<75 years vs. ≥75 years) and frailty status (non-frail vs. frail, based on a baseline modified Charlson Comorbidity Index score, patient age at point of study enrollment, and baseline Eastern Cooperative Oncology Group performance status score) [22] were also performed. All enrolled patients were included in the ITT population; patients who received ≥1 dose of IRd were included in the safety population.

## Results

### Patients and treatment

As of October 17, 2022 (the date of data accrual), 140 patients had been enrolled and treated (1 successfully screened patient was not treated) and were included in the safety and ITT populations. The median age of patients was 72.5 years; 59 patients (42.1%) were ≥75 years and 86 patients (61.4%) were considered frail (Table 1). Overall, 25 patients (17.9%) were Black or African American; 12 (8.6%) were Hispanic/Latino; 3 (2.1%) were Asian; and 1 (0.7%) was from the Pacific Islands. Of the entire cohort, 37 (26.4%), 58 (41.4%) and 44 (31.4%) patients had baseline disease stages I, II and III respectively, as defined by International Scoring System (ISS) criteria. Calculated baseline creatine clearance values among all patients were as follows: <60 mL/min, 40 patients (28.6%); ≥60 mL/min, 96 patients (68.6%); missing data, 4 patients (2.9%). At baseline, 131 patients (93.6%) had ≥1 concurrent medical condition, and 18 patients (12.9%) had peripheral neuropathy (PN). The most common induction regimens were bortezomib-lenalidomide-dexamethasone (VRd; 118 patients, 84.3%) and bortezomib-cyclophosphamide-dexamethasone (18 patients, 12.9%) (Table 1).

**Table 1:** Baseline demographics and disease characteristics.

		Age		Frailty status	
Characteristic	Overall (*N* = 140)	<75 years (*n* = 81)	≥75 years (*n* = 59)	Non-frail (*n* = 54)	Frail (*n* = 86)
**Median age,**^**a**^ **years (range)**	72.5 (48–90)	69.0 (48–74)	77.0 (75–90)	71.0 (49–78)	75.0 (48–90)
Aged <65 years (%)	29 (20.7)	29 (35.8)	0	16 (29.6)	13 (15.1)
Aged 65–<75 years (%)	52 (37.1)	52 (64.2)	0	26 (48.1)	26 (30.2)
Aged ≥75 years (%)	59 (42.1)	0	59 (100)	12 (22.2)	47 (54.7)
**Male,** ***n*** **(%)**	81 (57.9)	49 (60.5)	32 (54.2)	35 (64.8)	46 (53.5)
**Race,** ***n*** **(%)**					
White	102 (72.9)	57 (70.4)	45 (76.2)	40 (74.1)	62 (72.1)
Black/African American	25 (17.9)	15 (18.5)	10 (16.9)	8 (14.8)	17 (19.8)
Asian	3 (2.1)	2 (2.5)	1 (1.7)	1 (1.9)	2 (2.3)
Native Hawaiian or Other Pacific Islander	1 (0.7)	1 (1.2)	0	1 (1.9)	0
**Ethnicity,** ***n*** **(%)**^**b**^					
Hispanic/Latino	12 (8.6)	9 (11.1)	3 (5.1)	5 (9.3)	7 (8.1)
**ISS**^**c**^ **disease stage,** ***n*** **(%)**					
I / II / III	37 (26.4)/58 (41.4)/44 (31.4)	21 (25.9)/35 (43.2)/24 (29.6)	16 (27.1)/23 (39.0)/20 (33.9)	14 (25.9)/23 (42.6)/17 (31.5)	23 (26.7)/35 (40.7)/27 (31.4)
**Type of myeloma at initial diagnosis,** ***n*** **(%)**					
Heavy chain type					
IgG	83 (59.3)	48 (59.3)	35 (59.3)	29 (53.7)	54 (62.8)
IgA	30 (21.4)	19 (23.5)	11 (18.6)	13 (24.1)	17 (19.8)
IgE	0	0	1 (1.7)	0	0
IgM	1 (0.7)	0	1 (1.7)	0	1 (1.2)
IgD	1 (0.7)	1 (1.2)	0	0	1 (1.2)
Other	2 (1.4)	1 (1.2)	1 (1.7)	1 (1.9)	1 (1.2)
Multiple	10 (7.1)	6 (7.4)	4 (6.8)	6 (11.1)	4 (4.7)
Missing	13 (9.3)	6 (7.4)	7 (11.9)	5 (9.3)	8 (9.3)
Light chain type, *n* (%)					
Kappa	85 (60.7)	54 (66.7)	31 (52.5)	33 (61.1)	52 (60.5)
Lambda	42 (30.0)	17 (21.0)	25 (42.4)	15 (27.8)	27 (31.4)
Multiple	12 (8.6)	9 (11.1)	3 (5.1)	6 (11.1)	6 (7.0)
Missing	1 (0.7)	1 (1.2)	0	0	1 (1.2)
**Evidence of lytic bone disease,** ***n*** **(%)**					
Yes	64 (45.7)	38 (46.9)	26 (44.1)	29 (53.7)	35 (40.7)
No	64 (45.7)	35 (43.2)	29 (49.2)	21 (38.9)	43 (50.0)
Unknown	12 (8.6)	8 (9.9)	4 (6.8)	4 (7.4)	8 (9.3)
**Evidence of extramedullary disease,** ***n*** **(%)**					
Yes	10 (7.1)	7 (8.6)	3 (5.1)	6 (11.1)	4 (4.7)
No	105 (75.0)	59 (72.8)	46 (78.0)	38 (70.4)	67 (77.9)
Unknown	25 (17.9)	15 (18.5)	10 (16.9)	10 (18.5)	15 (17.4)
**Calculated CrCl,** ***n*** **(%)**					
<30 mL/min	5 (3.6)	3 (3.7)	2 (3.4)	0	5 (5.8)
30 to <60 mL/min	35 (25.0)	16 (19.8)	19 (32.2)	8 (14.8)	27 (31.4)
60 to <90 mL/min	57 (40.7)	26 (32.1)	31 (52.5)	25 (46.3)	32 (37.2)
>90 mL/min	39 (27.9)	36 (44.4)	3 (5.1)	21 (38.9)	18 (20.9)
Missing	4 (2.9)	0	4 (6.8)	0	4 (4.7)
**≥1 comorbidity at start of IRd therapy,** ***n*** **(%)**	131 (93.6)	74 (91.4)	57 (96.6)	50 (92.6)	81 (94.2)
**Induction regimen,** ***n*** **(%)**					
VRd	118 (84.3)	68 (84.0)	50 (84.7)	47 (87.0)	71 (82.6)
VCd	18 (12.9)	11 (13.6)	7 (11.9)	5 (9.3)	13 (15.1)
Other (Vd, VR)	4 (2.9)	2 (2.5)	2 (3.4)	2 (3.7)	2 (2.3)

*CrCl* creatinine clearance, *Ig* immunoglobuline, *IRd* ixazomib-lenalidomide-dexamethasone, *ISS* International Staging System, *VCd* bortezomib-cyclophosphamide-dexamethasone, *Vd* bortezomib-dexamethasone, *VR* bortezomib-lenalidomide, *VRd* bortezomib-lenalidomide-dexamethasone.

^a^Age and CrCl captured at start of IRd.

^b^No other patients were reported as Hispanic/Latino; 3 were not reported and 2 were unknown.

^c^ISS captured at start of bortezomib-based induction.

At data accrual, 14 patients (10.0%) were ongoing on IRd treatment, 111 (79.3%) had discontinued study treatment, and 12 (8.5%) had completed ≥39 cycles of IRd. Another 3 patients had completed 26 treatment cycles per the original study protocol. The most common reasons for treatment discontinuation were AEs (27 patients, 19.3%), patient withdrawal (26 patients, 18.6%), and PD (23 patients, 16.4%; Table 2). Overall, 3 patients (2.1%) underwent autologous stem-cell transplantation following IRd.

**Table 2:** Patient disposition^a^.

	Age			Frailty status		
	Overall (*N* = 140)	<75 years (*n* = 81)	≥75 years (*n* = 59)		Non-frail (*n* = 54)	Frail (*n* = 86)
**Treated subjects,** ***n*** **(%)**	140 (100.0)	81 (100.0)	59 (100.0)		54 (100.0)	86 (100.0)
Ongoing in treatment period	14 (10.0)	10 (12.3)	4 (6.8)		7 (13.0)	7 (8.1)
Completed study drug	15 (10.7)	11 (13.6)	4 (6.8)		2 (3.7)	13 (15.1)
Discontinued study drug	111 (79.3)	60 (74.1)	51 (86.4)		45 (83.3)	66 (76.7)
Ongoing in follow-up period	2 (1.4)	1 (1.2)	1 (1.7)		0	2 (2.3)
Completed follow-up period	47 (33.6)	28 (34.6)	19 (32.2)		21 (38.9)	26 (30.2)
Discontinued follow-up period	62 (44.3)	31 (38.3)	31 (52.5)		24 (44.4)	38 (44.2)
Ongoing in follow-up period	17 (12.1)	11 (13.6)	6 (10.2)		7 (13.0)	10 (11.6)
**Reasons for study drug discontinuation,** ***n*** **(%)**						
Adverse event	27 (19.3)	15 (18.5)	12 (20.3)		13 (24.1)	14 (16.3)
Unacceptable toxicity	1 (0.7)	1 (1.2)	0		1 (1.9)	0
Protocol deviation	0	0	0		0	0
Lost to follow-up	1 (0.7)	0	1 (1.7)		0	1 (1.2)
Withdrawal by patient	26 (18.6)	11 (13.6)	15 (25.4)		10 (18.5)	16 (18.6)
Progressive disease	23 (16.4)	13 (16.0)	10 (16.9)		5 (9.3)	18 (20.9)
Physician decision	14 (10.0)	9 (11.1)	5 (8.5)		8 (14.8)	6 (7.0)
Study terminated by sponsor	1 (0.7)	0	1 (1.7)		0	1 (1.2)
Other	18 (12.9)	11 (13.6)	7 (11.9)		8 (14.8)	10 (11.6)
**Completed study,** ***n*** **(%)**	61 (43.6)	39 (48.1)	22 (37.3)		23 (42.6)	38 (44.2)
**Discontinued study,** ***n*** **(%)**	62 (44.3)	31 (38.3)	31 (52.5)		24 (44.4)	38 (44.2)
Reason for study discontinuation						
Adverse event	6 (4.3)	4 (4.9)	2 (3.4)		2 (3.7)	4 (4.7)
Unacceptable toxicity	0	0	0		0	0
Protocol deviation	0	0	0		0	0
Lost to follow-up	2 (1.4)	0	2 (3.4)		0	2 (2.3)
Withdrawal by patient	17 (12.1)	7 (8.6)	10 (16.9)		8 (14.8)	9 (10.5)
Progressive disease	12 (8.6)	6 (7.4)	6 (10.2)		2 (3.7)	10 (11.6)
Physician decision	6 (4.3)	3 (3.7)	3 (5.1)		5 (9.3)	1 (1.2)
Study terminated by sponsor	0	0	0		0	0
Other	19 (13.6)	11 (13.6)	8 (13.6)		7 (13.0)	12 (14.0)

^a^Percentages are based on the number of patients in the safety population, except where indicated.

### Duration of treatment

#### Median duration of IRd

The overall median duration of all PI therapy (including the 3 cycles of bortezomib-based induction) was 13.6 months, while the overall median duration of IRd therapy was 11.0 months, with a median of 11 treated cycles (Table 3). Among patient subgroups aged <75 and ≥75 years, median IRd therapy duration was 13.8 months and 9.2 months, respectively, while the median number of treatment cycles was 13 and 9, respectively. Non-frail patients were treated with IRd for a median of 11.9 months with a median of 12 cycles, while frail patients were treated for a median of 10.3 months with a median of 11 cycles.

**Table 3:** Duration of treatment.

Variable	Treatment duration, months				
	Overall (*N* = 140)	<75 years (*n* = 81)	≥75 years (*n* = 59)	Non-frail (*n* = 54)	Frail (*n* = 86)
**PI therapy, including bortezomib-based induction**^**a**^					
Mean (StDev)	17.9 (12.2)	19.8 (12.8)	15.2 (10.8)	17.0 (11.1)	18.4 (12.8)
Median^b^	13.6	18.0	11.5	14.6	13.3
Range	3.0–41.2	3.0–41.2	3.2–40.3	3.1–39.3	3.0–41.2
**IRd**^**c**^					
Mean (StDev)	14.9 (12.3)	16.8 (12.9)	12.3 (11.0)	14.2 (11.1)	15.3 (13.1)
Median^b^	11.0	13.8	9.2	11.9	10.3
Range	0.7–38.0	0.7–37.8	0.7–38.0	0.7–36.6	0.7–38.0
**Ixazomib**^**d**^					
Mean (StDev)	14.5 (12.4)	16.5 (13.0)	11.8 (11.1)	13.7 (11.3)	15.0 (13.1)
Median^b^	10.5	13.2	8.5	10.8	10.1
Range	0.5–37.8	0.5–37.5	0.5–37.8	0.5–36.3	0.5–37.8

*IRd* ixazomib-lenalidomide-dexamethasone, *PI* proteasome inhibitor, *StDev* standard deviation.

^a^Duration of proteasome inhibitor therapy is defined as the time from the date of first administration of the bortezomib-based regimen to the date of the last administration of ixazomib.

^b^Simple median not calculated using the Kaplan-Meier method.

^c^Duration of IRd is defined as the time from the date of the first. administration of the study drug regimen (IRd) to the date of the last administration of any of the three study drugs in the regimen.

^d^Duration of ixazomib therapy is defined as the time from the date of first administration of ixazomib therapy to the date of the last administration of ixazomib therapy.

Overall, 102 patients (72.9%) completed five or more cycles of IRd; in the subgroups aged <75 and ≥75 years, 61 (75.3%) and 41 (69.5%) patients completed five or more cycles, respectively. Among non-frail and frail subgroups, 39 (72.2%) and 63 (73.3%) patients completed five or more cycles, respectively.

#### Median duration of ixazomib

The median duration of ixazomib therapy among the entire cohort was 10.5 months and the median duration of all PI-based therapy (including bortezomib-based induction) was 13.6 months. For patients aged <75 and ≥75 years, median durations of therapy with ixazomib were 13.2 and 8.5 months, respectively; for all PI-based therapy, median durations of therapy were 18.0 and 11.5 months, respectively. Among subgroups of non-frail and frail patients, median durations of ixazomib treatment were 10.8 and 10.1 months, while median durations of treatment of total PI-based therapy were 14.6 and 13.3 months, respectively (Table 3).

### Efficacy

**Fig. 1:**
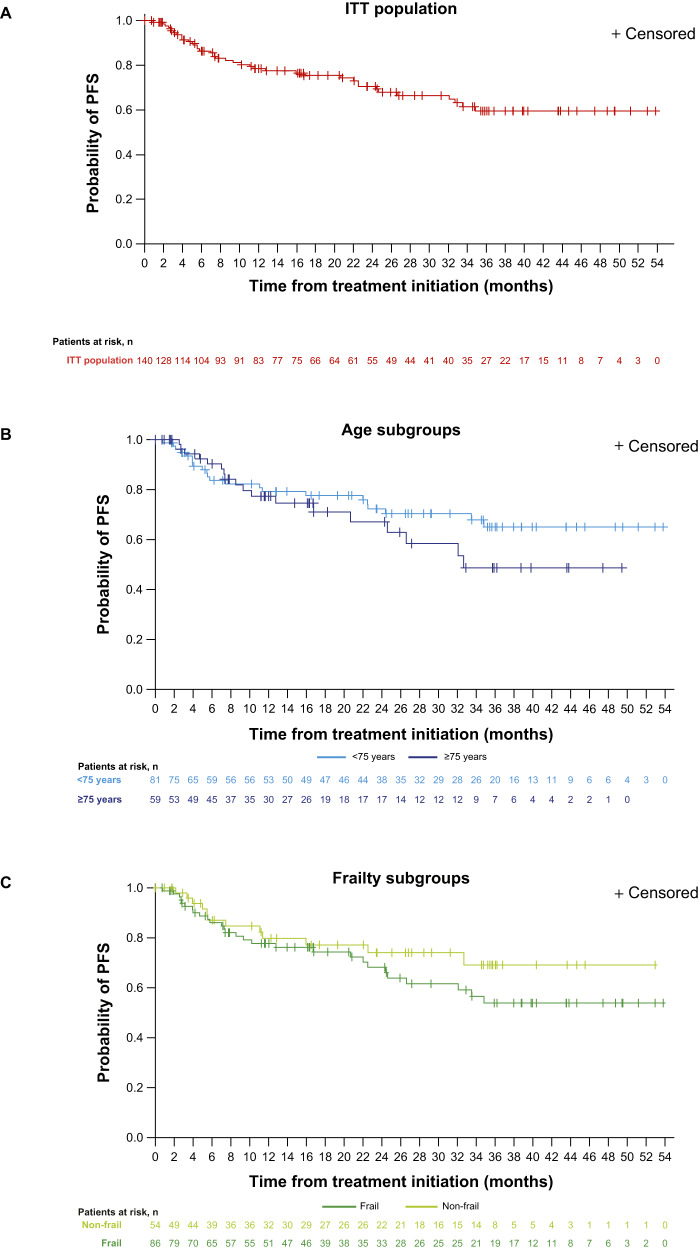
Investigator-assessed PFS from start of IRd. **A** ITT population (*N* = 140^a^). **B** Stratified by age subgroup. **C** Stratified by frailty subgroup. *IMWG* International Myeloma Working Group, *IRd* ixazomib-lenalidomide-dexamethasone, *ITT* intent-to-treat, *PD* progressive disease, *PFS* progression-free survival. ^a^One successfully screened patient was not treated. PFS defined as the time from first administration of IRd to the date of the first documentation of PD based on local laboratory results and the investigator’s assessment using modified IMWG response criteria, or death due to any cause, whichever occurred first; data are stratified by (**A**) ITT population; (**B**) subgroups aged <75 and ≥75 years and (**C**) subgroups defined as non-frail and frail.

After a median follow-up of 26.8 months at data accrual, 29 patients had progressed and 11 had died. In subgroups aged <75 and ≥75 years, median follow-up was 29.2 and 24.3 months, respectively; for non-frail and frail patients, median follow-up was 27.5 and 26.7 months, respectively. The overall 2-year PFS rate (KM estimate) from the start of IRd treatment was 71% (95% CI: 61–78), and median PFS had not been reached (Fig. 1A). Among subgroups of interest, for patients aged <75 and ≥75 years, 2-year PFS rates were 72% (95% CI: 61–81; Fig. 1B) and 67% (95% CI: 50–80; Fig. 1C). Median PFS values were not reached among patients aged <75 years, and 32.7 months (95% CI: 20.7–not calculable) among those aged ≥75 years. For patients defined as non-frail and frail, 2-year PFS rates were 74% (95% CI: 58–85) and 68% (95% CI: 55–78), respectively; for both subgroups, median PFS was not reached.

**Fig. 2:**
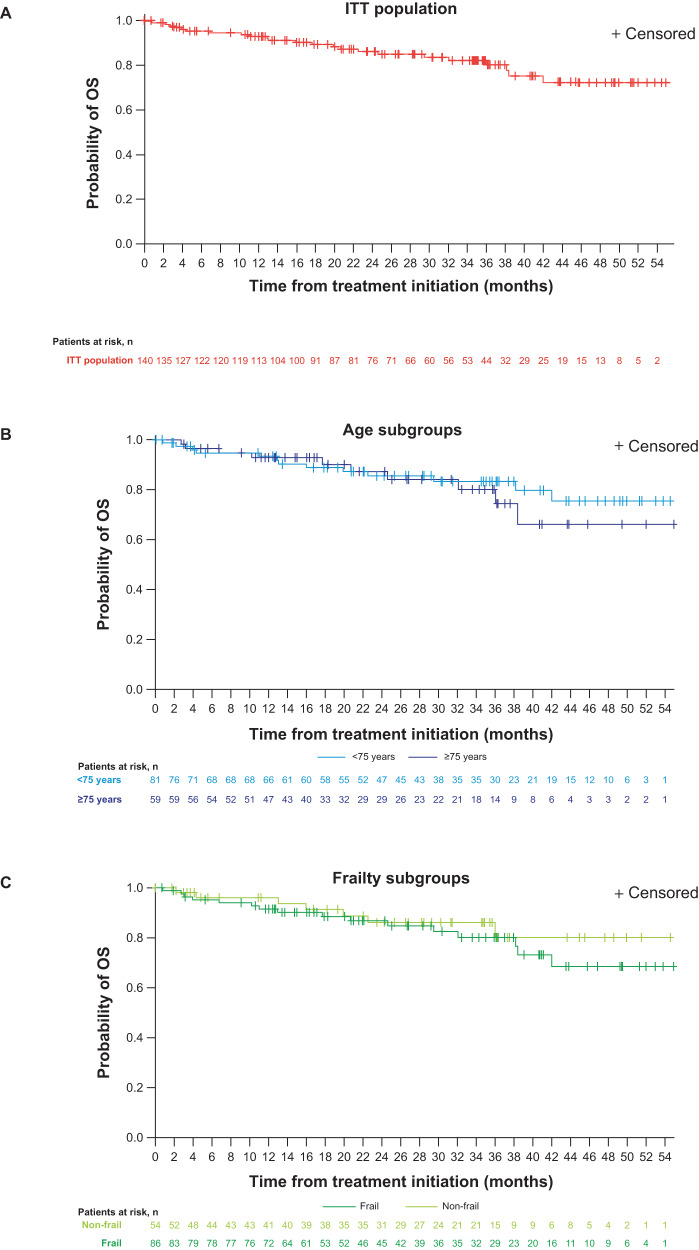
OS from start of IRd. **A** ITT population (*N* = 140^a^). **B** Stratified by age subgroup. **C** Stratified by frailty subgroup. *IRd* ixazomib-lenalidomide-dexamethasone, *ITT* intent-to-treat, *OS* overall survival. ^a^One successfully screened patient was not treated. OS defined as the time from the date of the first administration of IRd to the date of death from any cause. Patients without documentation of death at the time of analysis were censored at the date last known to be alive; data are stratified by (**A**) ITT population; (**B**) subgroups aged <75 and ≥75 years and (**C**) subgroups defined as non-frail and frail.

The overall 2-year OS rate (KM estimate) from the start of IRd treatment was 86% (Fig. 2A), 86% and 87%, for patients aged <75 years and ≥75 years (Fig. 2B) and 86% and 87%, for non-frail and frail patients (Fig. 2C), respectively. At data accrual, median OS had not been reached in either the overall population or in any of the subgroups.

**Fig. 3:**
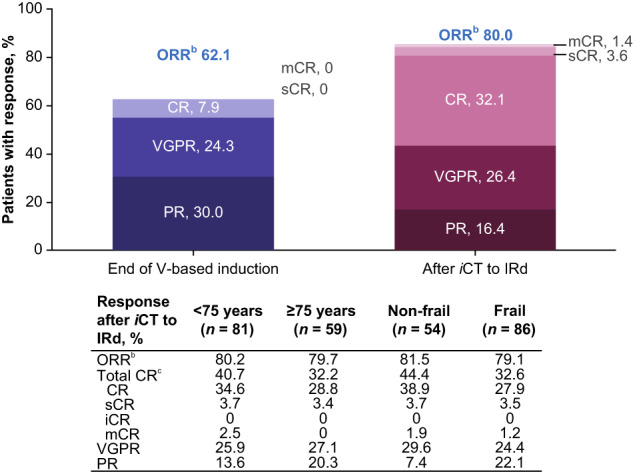
Response rates at the end of V-based induction and after *i*CT to IRd (ITT population; *N* = 140^a^). CR complete response, iCR immunophenotypic CR, *iCT* in-class transition, IRd ixazomib-lenalidomide-dexamethasone, ITT intent-to-treat, mCR molecular CR, *ORR* overall response rate, PR partial response, sCR stringent CR, *V* bortezomib, VGPR very good partial response. ^a^One successfully screened patient was not treated. ^b^ORR = PR + VGPR + CR + sCR + iCR + mCR. ^c^Total CR = CR + sCR + iCR + mC.

Among the entire cohort, overall response rate (ORR) had increased from 62.1% (CR 7.9%; very good partial response [VGPR] 24.3%; partial response [PR] 30.0%) at the end of bortezomib-based induction to 80.0% after *i*CT to IRd (CR [including stringent CR and molecular CR] 37.1%; VGPR 26.4%; PR 16.4%) (Fig. 3). For patients aged <75 years and ≥75 years, ORR increased from 60.5% and 64.4%, respectively, to 80.2% and 79.7% respectively following *i*CT. An increase in ORR from 70.4% to 81.5% was observed among non-frail patients, while an increase from 57.0% to 79.1% was observed among frail patients.

### Safety

Overall, 137 patients (97.9%) experienced any treatment emergent AE (TEAE); 96 patients (68.6%) had a grade ≥3 TEAE (Table 4). The most common TEAEs included gastrointestinal disorders and PNs. Gastrointestinal disorders were experienced by 93 patients (66.4%); of these, 60 patients (42.9%) experienced treatment-related gastrointestinal disorders. PNs were observed among 39 patients (27.9%, Table 5); 30 patients (21.4%) experienced treatment-related PNs. Overall, 36 patients (25.7%) had grades 1–2 PNs and 3 (2.1%) experienced grade 3 PNs; there were no cases of grade 4 PNs.

**Table 4:** Overview of the safety profile of IRd (safety population, *N* = 140).

		Age		Frailty status	
TEAEs, *n* (%)	Overall (*N* = 140)	<75 years (*n* = 81)	≥75 years (*n* = 59)	Non-frail (*n* = 54)	Frail (*n* = 86)
**Any TEAE**	137 (97.9)	79 (97.5)	58 (98.3)	52 (96.3)	85 (98.8)
Treatment-related	115 (82.1)	69 (85.2)	46 (78.0)	47 (87.0)	68 (79.1)
**Grade** ≥**3 TEAEs**	96 (68.6)	56 (69.1)	40 (67.8)	33 (61.1)	63 (73.3)
Treatment-related	52 (37.1)	27 (33.3)	25 (42.4)	19 (35.2)	33 (38.4)
**Serious TEAEs**	62 (44.3)	34 (42.0)	28 (47.5)	21 (38.9)	41 (47.7)
Treatment-related	18 (12.9)	10 (12.3)	8 (13.6)	8 (14.8)	10 (11.6)
**TEAE leading to drug modification**^**a**^	93 (66.4)	54 (66.7)	39 (66.1)	33 (61.1)	60 (69.8)
**TEAE leading to drug discontinuation**^**a**^	28 (20.0)	16 (19.8)	12 (20.3)	14 (25.9)	14 (16.3)
**On-study deaths**^**b**^	4 (2.9)	2 (2.5)	2 (3.4)	1 (1.9)	3 (3.5)

*IRd* ixazomib-lenalidomide-dexamethasone, *TEAE* treatment emergent adverse event.

^a^Modifications and discontinuations for any of the three study drugs.

^b^Occurring <30 days after last dose; deaths were due to unrelated end-stage renal disease, treatment-related pneumonia, disease-related complications, and unknown (*N* = 1 each).

**Table 5:** Most commonly occurring treatment emergent adverse events^a^ (safety population; *N* = 140).

		Age		Frailty status	
TEAEs, *n* (%)	Overall (*N* = 140)	<75 years (*n* = 81)	≥75 years (*n* = 59)	Non-frail (*n* = 54)	Frail (*n* = 86)
**Diarrhea**	71 (50.7)	40 (49.4)	31 (52.5)	27 (50.0)	44 (51.2)
Grade 1–2	58 (41.4)	34 (42.0)	24 (40.7)	20 (37.0)	38 (44.2)
Grade ≥3	13 (9.3)	6 (7.4)	7 (11.9)	7 (13.0)	6 (7.0)
**PN**	39 (27.9)	24 (29.6)	15 (25.4)	12 (22.2)	27 (31.4)
Grade 1–2	36 (25.7)	22 (27.2)	14 (23.7)	10 (18.5)	26 (30.2)
Grade ≥3	3 (2.1)	2 (2.5)	1 (1.7)	2 (3.7)	1 (1.2)
**Fatigue**	47 (33.6)	29 (35.8)	18 (30.5)	15 (27.8)	32 (37.2)
Grade 1─2	42 (30.0)	27 (33.3)	15 (25.4)	12 (22.2)	30 (34.9)
Grade ≥3	5 (3.6)	2 (2.5)	3 (5.1)	3 (5.6)	2 (2.3)
**Nausea**	35 (25.0)	23 (28.4)	12 (20.3)	11 (20.4)	24 (27.9)
Grade 1–2	31 (22.1)	22 (27.2)	9 (15.3)	11 (20.4)	20 (23.3)
Grade ≥3	4 (2.9)	1 (1.2)	3 (5.1)	0	4 (4.7)
**Peripheral edema**	36 (25.7)	23 (28.4)	13 (22.0)	14 (25.9)	22 (25.6)
Grade 1–2	35 (25.0)	23 (28.4)	12 (20.3)	14 (25.9)	21 (24.4)
Grade ≥3	1 (0.7)	0	1 (1.7)	0	1 (1.2)
**Arthralgia**	31 (22.1)	21 (25.9)	10 (16.9)	11 (20.4)	20 (23.3)
Grade 1–2	28 (20.0)	19 (23.5)	9 (15.3)	10 (18.5)	18 (20.9)
Grade ≥3	3 (2.1)	2 (2.5)	1 (1.7)	1 (1.9)	2 (2.3)
**Back pain**	26 (18.6)	17 (21.0)	9 (15.3)	8 (14.8)	18 (20.9)
Grade 1–2	23 (16.4)	15 (18.5)	8 (13.6)	8 (14.8)	15 (17.4)
Grade ≥3	3 (2.1)	2 (2.5)	1 (1.7)	0	3 (3.5)
**Constipation**	24 (17.1)	12 (14.8)	12 (20.3)	7 (13.0)	17 (19.8)
Grade 1–2	24 (17.1)	12 (14.8)	12 (20.3)	7 (13.0)	17 (19.8)
Grade ≥3	0	0	0	0	0
**Hypokalemia**	25 (17.9)	12 (14.8)^b^	13 (22.0)	3 (5.6)	22 (25.6)^b^
Grade 1–2	19 (13.6)	10 (12.3)	9 (15.3)	2 (3.7)	17 (19.8)
Grade ≥3	6 (4.3)	2 (2.5)	4 (6.8)	1 (1.9)	5 (5.8)
**Rash**	24 (17.1)	14 (17.3)	10 (16.9)	7 (13.0)	17 (19.8)
Grade 1–2	20 (14.3)	11 (13.6)	9 (15.3)	6 (11.1)	14 (16.3)
Grade ≥3	4 (2.9)	3 (3.7)	1 (1.7)	1 (1.9)	3 (3.5)
**Pneumonia**	18 (12.9)	10 (12.3)	8 (13.6)	6 (11.1)	12 (14.0)
Grade 1–2	9 (6.4)	7 (8.6)	2 (3.4)	5 (9.3)	4 (4.7)
Grade ≥3	9 (6.4)	3 (3.7)	6 (10.2)	1 (1.9)	8 (9.3)

*PN* peripheral neuropathy, *TEAE* treatment emergent adverse event.

^a^In >15% of patients at any grade or >5% at grade ≥3. None of the TEAEs in this table were grade 4, except where indicated.

^b^1 patient aged <75 years, deemed frail, had grade 4 hypokalemia.

### Patient-reported quality of life, treatment satisfaction, and global satisfaction

Of all 1875 EORTC QLQ-C30, EORTC QLQ-MY20, and TSQM-9 questionnaires that could have been completed in the entire sample, 1004 (53.5%), 1012 (54.0%), and 1012 (54.0%) had been completed by patients, respectively. Considering that only a subset of all possible questionnaires were successfully launched to patients, the overall completion percentage was re-calculated as 95.2%.

Overall, patient-reported QoL (EORTC QLQ-C30 GHS/QoL) was maintained during IRd therapy in the overall cohort (Supplementary Fig. 1A), and by age and frailty status (Supplementary Fig. 1B). Treatment satisfaction (TSQM-9 Effectiveness [Supplementary Fig. 2A], Treatment Convenience [Supplementary Fig. 2B], and Global Satisfaction with treatment [Supplementary Fig. 2C]) was also generally maintained during IRd therapy among the overall cohort, and by age and frailty status. For item 43 in the EORTC QLQ-MY20 questionnaire measuring the burden of PN [23], the mean change from ePRO baseline score was ≤0.7 during IRd treatment (Supplementary Fig. 3A), while mean changes from ePRO baseline scores in age and frailty subgroups were similarly minor (Supplementary Fig. 3B). For all ePRO outcomes, considering the relatively small sample sizes which decreased over time, results were interpreted with caution at later cycles and for all subgroups.

### Actigraphy

Among 94 patients with available daily actigraphy data (a total of 26,665 days), 24,283 (91.1%) compliant days were included in the analysis. The mean number of steps per day was 3107 (StDev 2360). Mean daily active time among the whole cohort was 25.2 minutes (StDev 19.8), while for subgroups aged <75 and ≥75 years it was 25.8 (StDev 20.4) and 23.4 (StDev 17.4) minutes, respectively. Non-frail and frail patients were active for a mean of 27.0 (StDev 19.8) and 22.8 (StDev 19.2) minutes per day, respectively. These data are shown by cycle in Supplementary Fig. 4A. Mean daily sleep time for all patients was 7.6 hours (StDev 2.7). Patients aged <75 and ≥75 years slept for a mean of 7.8 (StDev 2.8) and 6.9 (StDev 2.4) hours daily, while non-frail and frail subgroups had mean daily sleep durations of 7.5 (StDev 2.5) and 7.6 (StDev 2.9) hours, respectively. These data are shown by cycle in Supplementary Fig. 4B.

## Discussion

These data from the fully accrued US MM-6 community-based cohort confirm preliminary findings, including promising overall 2-year PFS and OS rates of 71% and 86% respectively, high response rates and a deepened response after *i*CT to IRd (overall ORR increased from 62.1% at induction to 80.0% after *i*CT to IRd; total CR increased from 7.9% to 37.1%), a tolerable safety profile, with maintained QoL and actigraphy data [18]. Notably, the US MM-6 2-year PFS rate appears higher than figures typically reported by both RCTs and community-based studies investigating bortezomib-based regimens among transplant-ineligible patients with NDMM. For example, the community-based UPFRONT trial, which was designed to compare eight 21-day cycles of three bortezomib-based regimens (bortezomib-dexamethasone, bortezomib-dexamethasone-thalidomide and bortezomib-melphalan-prednisone [VMP]) followed by bortezomib maintenance, reported 2-year PFS values ranging from ~25–40% [20]. Additionally, the VISTA phase 3 RCT reported 2-year PFS rates of ~50% among patients with NDMM, who were administered nine planned 6-week cycles of VMP [4]. The phase 3 SWOG S0777 study reported a 2-year PFS of ~65% for previously untreated patients without an intent for immediate transplant, who received eight planned 21-day cycles of VRd followed by lenalidomide maintenance [6]. A phase 2 trial which administered nine planned 35-day cycles of modified VRd (RVD lite), followed by lenalidomide consolidation, in patients with previously untreated NDMM (NCT01782963) demonstrated a 2-year PFS of ~80%; however, the relatively small cohort size (*N* = 53) limits the study relevance to US MM-6; we also note that NCT01782963 was conducted at academic and medical centers, unlike the current study which was community-based [24]. The 2-year OS rate of US MM-6 was promising at 86%, and in line with TOURMALINE-MM2 outcomes for continuous ixazomib in transplant ineligible patients [17], and similar to cohorts of patients with NDMM who were administered bortezomib-based regimens (e.g., SWOG S0777, ~90% [6]; UPFRONT, ~75–80% [20]; VISTA, ~85% [4]). At data accrual, median OS had not been reached among the overall US MM-6 population, nor among any of the subgroups. While SWOG S0777 and US MM-6 demonstrated similar overall 2-year PFS and OS rates, it is notable that, compared to US MM-6, SWOG S0777 included generally younger and healthier patients, including patients who went on to receive transplant. Therefore, the SWOG S0777 patient population was likely not representative of MM populations typically seen in routine clinical practice; indeed, 39% of patients enrolled in SWOG S0777 were aged ≥65 years in the study arm, lower than the 79.3% of patients enrolled in the same age group of this real-world study.

As described above, ORR increased notably from the end of bortezomib-based induction to *i*CT to IRd. Similar increases were observed among subgroups aged <75 years and ≥75 years, as well as among non-frail and frail patients. The reported ORR, achieved with *i*CT among community-based US MM-6 patients, appears similar to the ORRs reported in the TOURMALINE-MM2 RCT (82.1%) [17] and the NCT01782963 (RVD lite) study (86%) [25]. The VISTA RCT reported an ORR of 70.6% among the VMP treatment arm [4], while UPFRONT reported ORRs of 69.7–79.7%, depending on the bortezomib-based regimen administered [20]. Deeper responses are key to prolonging PFS, particularly among patients with NDMM [26]. As noted, US MM-6 also demonstrated an overall deep response; total CR was 37.1% (CR 32.1%; stringent CR 3.6%; molecular CR 1.4%; immunophenotypic CR 0) in the ITT population following *i*CT to IRd, and was similar among subcohorts of interest. These rates are higher than corresponding rates from similar patient cohorts administered bortezomib-based treatment (SWOG S0777, CR 24.2% [6]; UPFRONT, CR 3–4% [20]; VISTA, CR 33% [4]). As opposed to many treatment regimens currently used for NDMM, US MM-6 did not include a maintenance component. Historically, maintenance was administered following transplant in MM to prolong the deep response obtained after transplantation. Maintenance treatment was later extended to transplant-ineligible patients receiving standard MM therapy. Use of a tolerable PI-based 3-drug regimen until progression results in a prolonged consolidation approach, which can facilitate maintenance of QoL and performance status. The median ixazomib treatment duration for patients aged <75 years was longer (13.2 months) than for patients aged ≥75 years (8.5 months), which could be due to the difference in study drug discontinuation rates (74.1% vs. 86.4%; including withdrawal by patient, 13.6% vs. 25.4%). However, the median duration of ixazomib treatment among the entire cohort was 10.5 months and similar durations were observed among subgroups of non-frail (10.8 months) and frail (10.1 months) patients, suggesting that long-term oral administration of IRd is viable and does not adversely impact the frail population. Furthermore, the median overall duration of PI therapy, including three cycles of bortezomib-based induction, was 13.6 months. In the SWOG S0777 study, patients were randomized to complete induction consisting of six 28-day cycles of Rd or eight 21-day cycles of VRd, followed by Rd maintenance [6]. While median cycles of study treatment have not been reported, only 55.7% (*n* = 131) of analyzable patients randomized to receive VRd completed induction. In addition, as mentioned above, patients tended to be younger compared with US MM-6 (39% in the study arm were aged ≥65 years vs 79.3% in US MM-6) [6]. For the current study, subgroup treatment durations generally support the tolerable safety profile of IRd in elderly and frail patients, permitting extended duration of treatment. Indeed, the overall rate of study drug discontinuation due to TEAEs in US MM-6 was 19.3%; this is lower than the equivalent rates reported in the community-based UPFRONT study (29–38%) [20]. Additionally, reviews analyzing real-world studies suggest that TEAEs are typically a leading cause of cancer therapy discontinuation (MM, ~16–39%; all cancer, ~15–32%) [12, 27] but are less prevalent in RCTs (MM: ~7–21%) [12, 28]. Of note, 18.6% of patients withdrew from treatment in US MM-6 but reasons for this were not collated. The proportion of patients choosing to withdraw is slightly lower than that reported in a retrospective analysis of 340 patients with MM who had received maintenance therapy (most commonly with bortezomib or lenalidomide) for <3 years post-autologous stem cell transplantation. In that study, 22.5% of patients discontinued due to patient preference. We acknowledge that non-collation of reasons for patient withdrawal is a limiting factor in our study and as such are currently updating the study database to enable capture of these missing data. We also demonstrate that the overall safety profile of IRd in US MM-6 was tolerable and similar to previous reports [17, 29]. Notably, 12.9% of patients had comorbid PN at baseline. Following *i*CT to IRd, PNs were experienced by 27.9% of patients; treatment-related PNs were experienced by 31.4%. Previous trials investigating bortezomib-based treatment among comparable transplant-ineligible patients with MM have generally demonstrated higher rates of PN occurrence (44–60%) [4, 20], suggesting that *i*CT to IRd could provide a tolerable treatment alternative. Mean EORTC QLQ-MY20 score was generally maintained during IRd treatment although for all ePRO outcomes, caution should be taken when interpreting later cycle data from patients aged ≥75 years, due to small cohort sizes. No adverse impact of *i*CT to IRd on QoL or treatment satisfaction was observed, and patients receiving IRd achieved activity levels (steps per day) and sleep durations comparable to previously published data [30, 31]. This should be considered impactful, as the overall cohort comprises a high proportion of elderly and frail patients, almost all with ≥1 comorbidity, among whom a decline in activity and QoL might be expected over the course of treatment.

The inclusion of MM patients from a real-world population in US MM-6 continues to lend credence to its findings [18]. Up to 40% of real-world patients with NDMM do not meet eligibility criteria for RCTs [32–34]. Eligible individuals are typically younger and have better health at baseline, and thus should not be considered entirely representative of all patients with MM. To address this, US MM-6 inclusion criteria were broader, allowing enrollment of patients who might not have access to RCTs, or who would typically be excluded due to comorbidities, making the enrolled population more representative of the US MM population. For example, Black or African American people constitute ~20% of the US population affected by MM [35], and US MM-6 included 17.9% Black or African American patients, whereas the typical RCT includes ~10% of Black or African American patients [36]. Patients had a median age of 72.5 years, 61.4% of patients were frail, and 31.4% of patients had an ISS score of III; all of these factors likely contributed to difficulties in tolerating treatment [37]. These patient baseline characteristics are similar to those reported in other community-based MM studies [20, 38, 39]. Nonetheless, in MM-6, comparable OS, PFS, and safety data between patients aged ≥75 years and frail patients versus the ITT population suggests that *i*CT to IRd is feasible for elderly and frail patients, in line with previous reports of the safety profile of ixazomib among elderly [40, 41] and frail NDMM patients [41].

Notably, US MM-6 did not include a comparator arm, making it difficult to evaluate the impact of *i*CT to IRd versus staying on bortezomib-based treatment. To address this, a comparative effectiveness analysis of the US MM-6 cohort versus a real-world bortezomib-based cohort from the multi-center, prospective, observational INSIGHT MM study was conducted [42]. The analysis indicated increased ORR, duration of treatment, and 2-year PFS for the *i*CT to IRd arm versus the continuous bortezomib-based cohort [42]. Real-world outcomes cannot be directly compared to RCT data; however, assessment of outcomes reported here, in the context of RCTs and community-based studies, suggests a clinical benefit for patients who undergo *i*CT to IRd and a manageable safety profile, which may be more practical for patients treated in the community.

US MM-6 may encourage reconsideration of patient eligibility criteria, as well as the use of the clinical standard of care at a given medical center for disease management and follow-up. It might also inform whether community-based oncology centers and patients are important to include, alongside academic centers, in meeting new Food and Drug Administration enrollment guidelines on diversity, equity, and inclusion in RCTs [43].

While *i*CT has rarely been studied in an RCT setting, it allows rapid disease reduction via a short-term regimen of parenteral bortezomib-based therapy, followed by long-term tolerable consolidation with an all-oral regimen. US MM-6 has demonstrated potential for sequential deepening of response following *i*CT, without seriously impacting QoL. This is important among community-based patients, for whom many therapeutics demonstrate reduced effectiveness and tolerability compared with published RCT data, likely due to the inherent differences in patient demographics described in this report and patient-specific environmental factors typically unaccounted for in RCTs.

US MM-6 enrollment lasted from November 2017 to May 2021; during the COVID pandemic, enrollment continued more slowly and enrolled patients continued according to study protocol. Many clinical studies were suspended during this period due to lack of cancer center access, but the US MM-6 oral triplet regimen, along with ePRO capture and teleconsultations, were amenable to uninterrupted treatment. Various guidelines suggested that patients should be transitioned to oral regimens during this period to continue therapy [44–47].

Current *i*CT trials are underway to investigate additional ixazomib-based treatment combinations among patients with MM; these include an RCT (NCT03942224) and others among patients with RRMM (NCT03416374, NCT03763162). It is expected that *i*CT will prove useful for other therapeutic approaches where long-term consolidation is important, with a focus on tolerable TEAE management.

In conclusion, updated outcomes of the fully accrued study cohort from the US MM-6 phase 4 study indicate promising PFS and OS data for community-based NDMM patients who undergo *i*CT from bortezomib-based induction to IRd treatment. ORRs were elevated following *i*CT, while actigraphy and PRO results suggest no adverse impact or decline in activity or QoL with continued treatment, and safety data suggest IRd is generally well tolerated. Furthermore, *i*CT from parenteral bortezomib-based induction to all-oral IRd permits long-term PI-based therapy translating into improved efficacy and outcomes in these underserved patients who are elderly, comorbid, who may not have access to an RCT or are unable to travel to an academic center or treatment site.

### Supplementary information

Supplementary Data

## Data Availability

The datasets, including the redacted study protocol, redacted statistical analysis plan, and individual participants’ data supporting the results reported in this article, will be made available from the completed study within three months from initial request, to researchers who provide a methodologically sound proposal. The data will be provided after its de-identification, in compliance with applicable privacy laws, data protection and requirements for consent and anonymization.
