# Supplementary material for: In-class transition (iCT) of proteasome inhibitor-based therapy: a community approach to multiple myeloma management
Source: Blood Cancer J. 2023 Sep 19;13(1):147. doi: 10.1038/s41408-023-00912-9 (PMC10509188; doi:10.1038/s41408-023-00912-9)
Supplement: Supplementary file 1 — Supplementary Data [file 41408_2023_912_MOESM1_ESM.pdf]

Supplementary Data

Supplementary Fig S1. Mean change from baseline in EORTC QLQ-C30 GHS/QoL per cycle. A ITT population (N = 140[a]). **B** Stratified by subgroup.

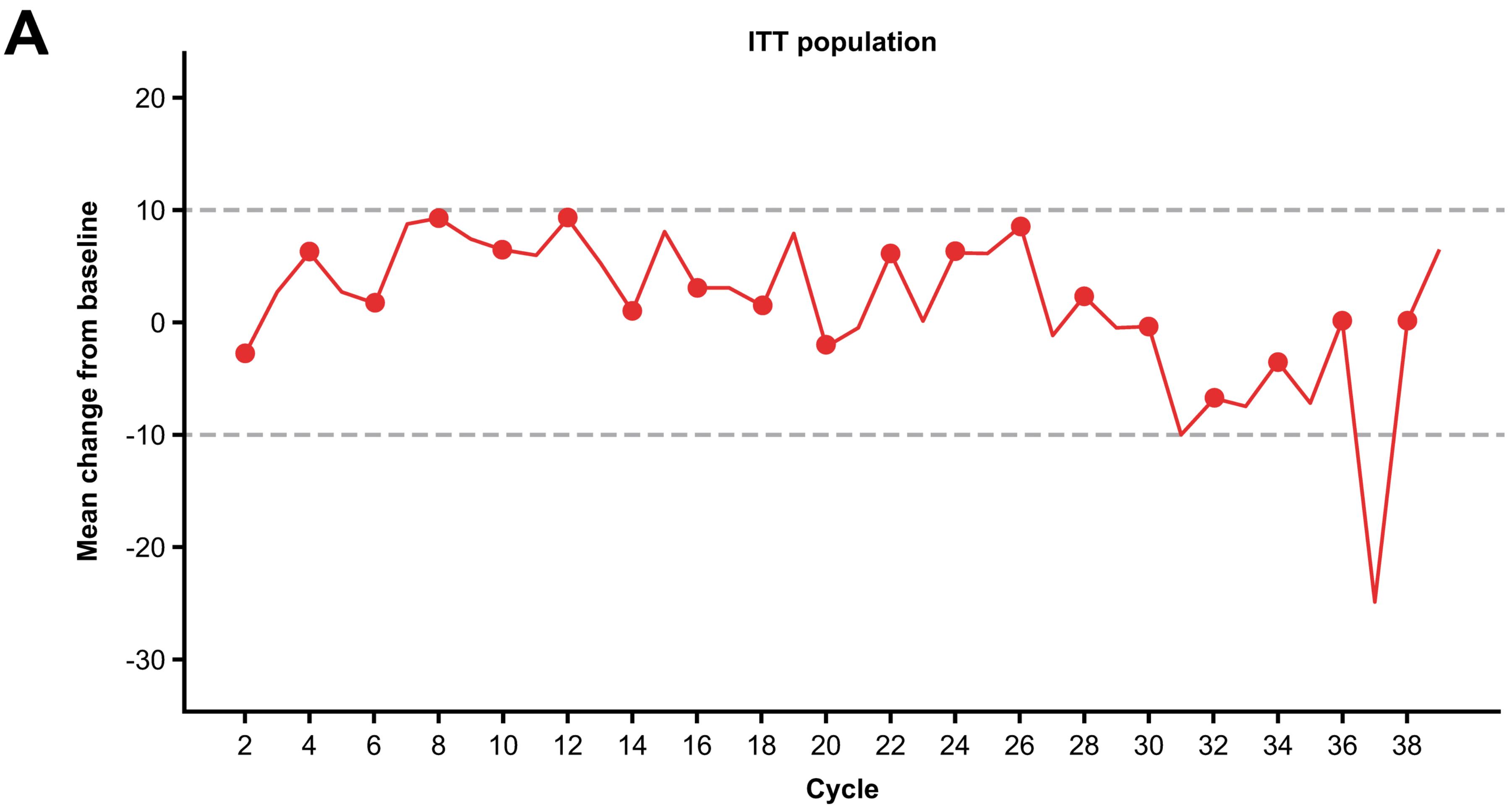

Patients, *n*

	2	4	6	8	10	12	14	16	18	20	22	24	26	28	30	32	34	36	38
ITT population	47	39	33	27	27	20	20	19	17	15	17	16	12	16	12	11	12	6	7

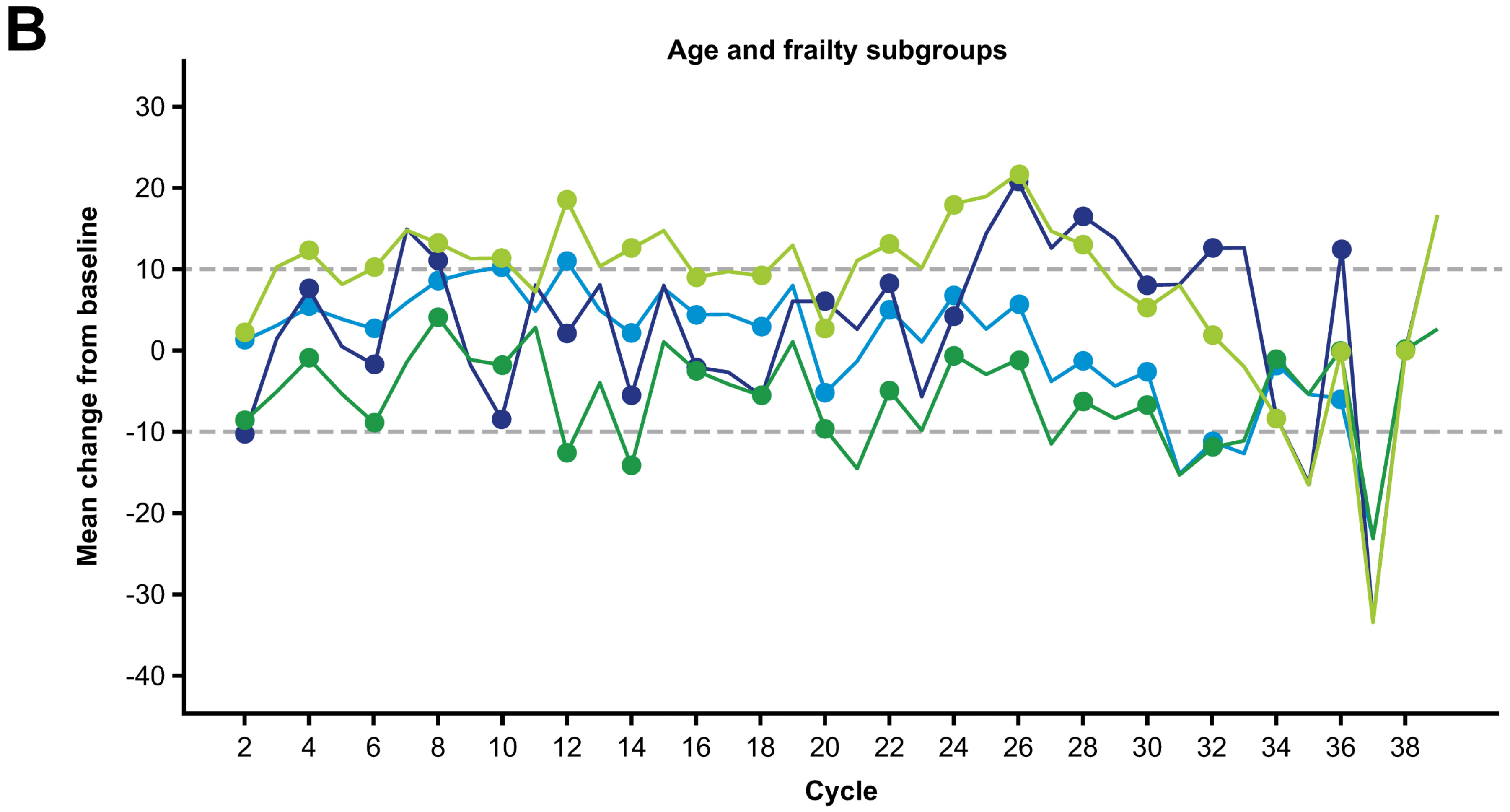

Patients, *n*

	2	4	6	8	10	12	14	16	18	20	22	24	26	28	30	32	34	36	38
<75 years	30	27	24	21	21	16	17	15	14	11	13	12	10	13	10	9	9	4	6
≥75 years	17	12	9	6	6	4	3	4	3	4	4	4	2	3	2	2	3	2	1
Non-frail	25	21	18	15	16	14	11	9	8	9	10	6	5	7	6	4	4	1	1
Frail	22	18	15	12	11	6	9	10	9	6	7	10	7	9	6	7	8	5	6

EORTC QLQ-C30 European Organisation for Research and Treatment of Cancer quality of life questionnaire, *ePRO* electronic patient reported outcome, *GHS* Global Health Status, *IRd* ixazomib-lenalidomide-dexamethasone, *ITT* intent-to-treat, *QoL* quality of life.

[a]One successfully screened patient was not treated. The EORTC QLQ-C30 GHS/QoL (derived from items 29 and 30 of the EORTC QLQ-C30, version 3) scale has a range of 0 to 100; positive and negative changes indicate improvement and deterioration in QoL, respectively. ePRO baseline defined as the reported measurement at the end of cycle 1 of IRd. Change from ePRO baseline only calculated at post-ePRO baseline IRd cycles where a value was present, and among patients with an ePRO baseline value. Data available up to 39 cycles. Data are stratified by **(A)** ITT population and (**B**) subgroup (patients aged <75 and ≥75 years, patients deemed non-frail and frail).

Supplementary Fig S2. Mean change from baseline in TSQM-9 subscale scores per cycle. A Effectiveness. **B** Convenience. **C** Global satisfaction

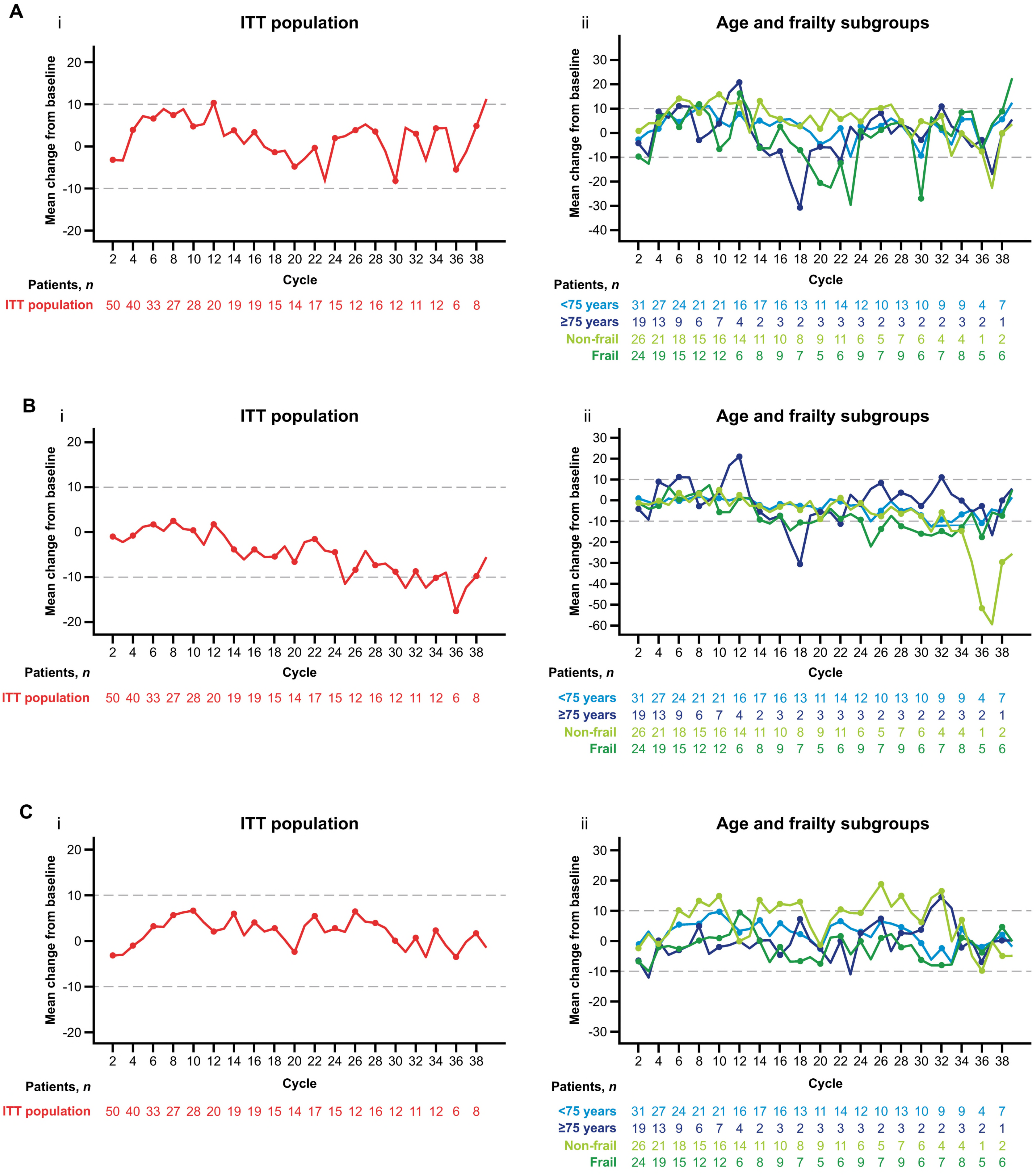

ePRO electronic patient reported outcomes, *IRd* ixazomib-lenalidomide-dexamethasone, *ITT* intent-to-treat, *TSQM-9* Treatment Satisfaction Questionnaire for Medication-9.

ePRO baseline defined as the reported measurement at the end of cycle 1 of IRd. Change from ePRO baseline only calculated at post-ePRO baseline IRd cycles where a value was present, and among patients with an ePRO baseline value TSQM-9 subscale scores measure the treatment effectiveness (**A**), convenience (**B**), and global satisfaction (**C**). These scales have a range of 0 to 100 (higher is better).

Supplementary Fig S3. Mean change from peripheral neuropathy baseline score in EORTC Multiple Myeloma Module (QLQ-MY20) per cycle. A ITT population (*N* = 140[a]). **B** Stratified by subgroup.

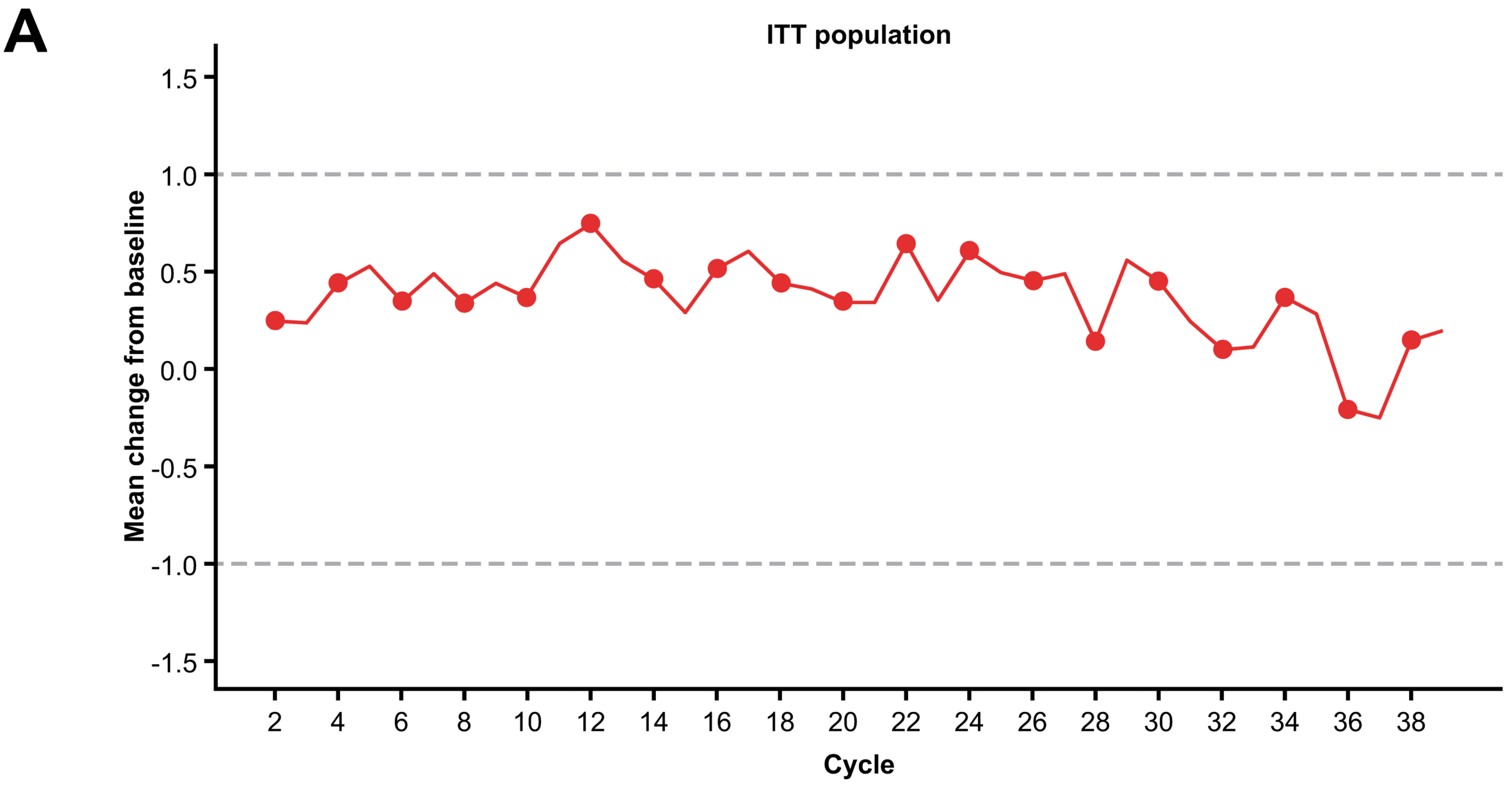

**Patients, *n***																			
**ITT population**	48	39	32	26	26	19	19	19	16	14	17	15	11	15	11	10	11	5	7

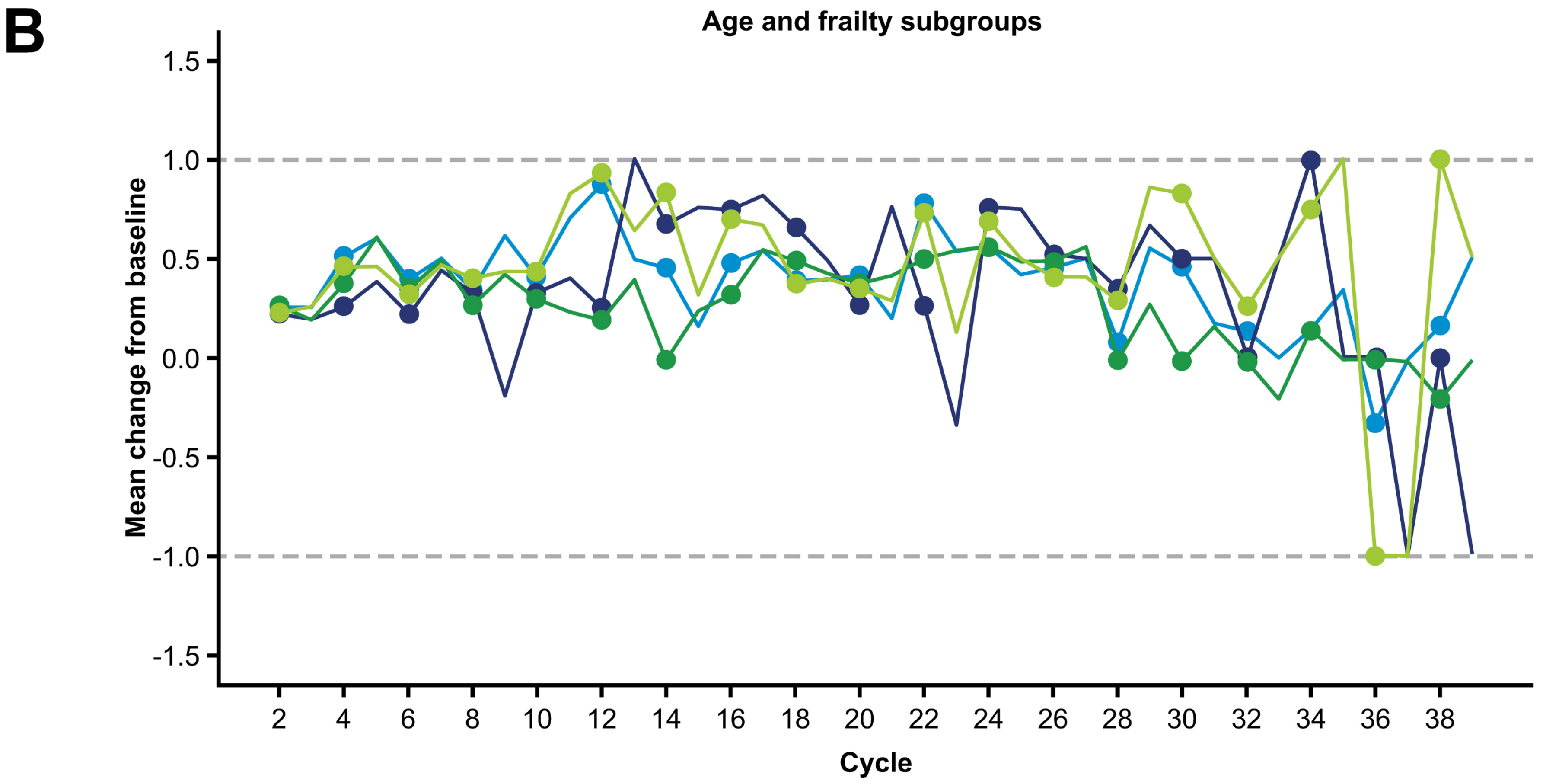

**Patients, *n***																			
**<75 years**	31	27	23	20	20	15	16	15	13	10	13	11	9	12	9	8	8	3	6
**≥75 years**	17	12	9	6	6	4	3	4	3	4	4	4	2	3	2	2	3	2	1
**Non-frail**	26	21	18	15	16	14	11	10	8	9	11	6	5	7	6	4	4	1	2
**Frail**	22	18	14	11	10	5	8	9	8	5	6	9	6	8	5	6	7	4	5

EORTC European Organisation for Research and Treatment of Cancer, *ePRO* electronic patient reported outcomes, *IRd* ixazomib-lenalidomide-dexamethasone, *ITT* intent-to-treat, *QLQ-MY20* Quality of life questionnaire MM module.

[a]One successfully screened patient was not treated. ePRO baseline defined as the reported measurement at the end of cycle 1 of IRd. Change from ePRO baseline only calculated at post-ePRO baseline IRd cycles where a value was present, and among patients with an

ePRO baseline value. EORTC QLQ-MY20, European Organization for Research and Treatment of Cancer Quality of Life Questionnaire Multiple Myeloma module 20 – item 43 measuring peripheral neuropathy, on a scale from 0 to 100. EORTC QLQ-MY20 item 43 (“did you have tingling hands or feet?”) measured the burden of peripheral neuropathy symptoms using a score range of 1 to 4 (1, not at all; 2, a little; 3, quite a bit; and 4, very much). A higher score indicates an increase in symptoms. Data are stratified by **(A)** ITT population and (**B**) subgroup (patients aged <75 and ≥75 years, patients deemed non-frail and frail).

Supplementary Fig S4. Actigraphy outcomes. A Mean daily active time per cycle[a]. **B** Mean daily sleep time per cycle[b].

A

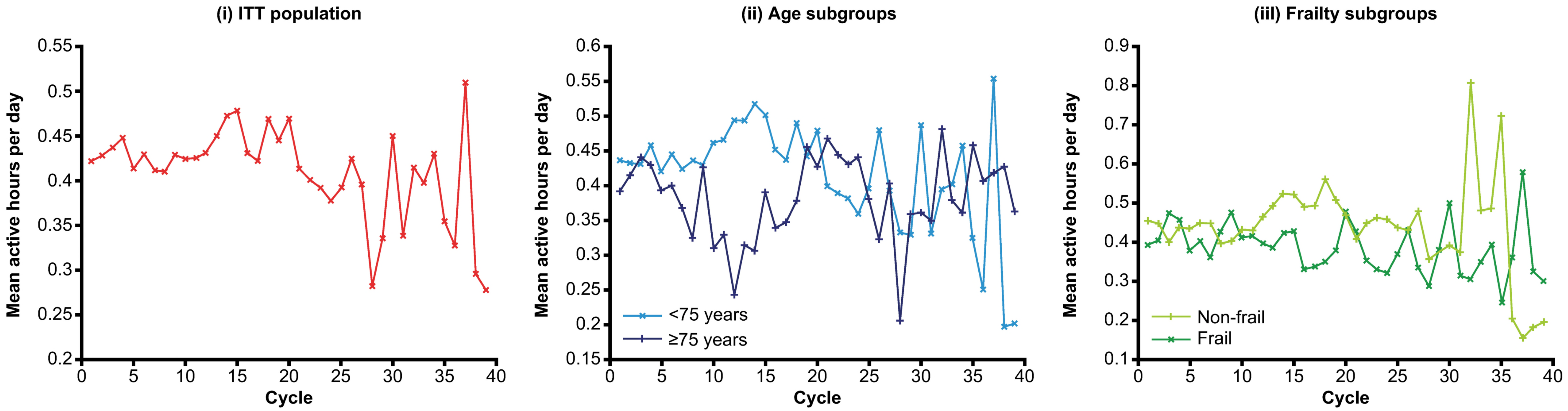

B

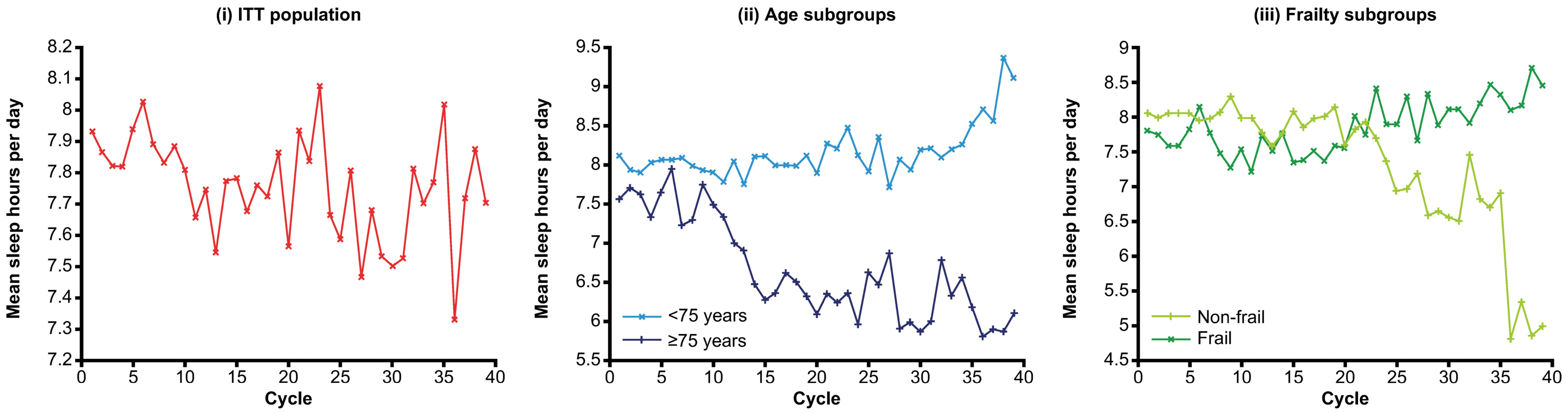

ITT intent-to-treat.

[a]Active time defined as the time for which patients were ‘active’ or ‘highly active’.

[b]Sleep time includes deep sleep; light sleep; “awake” time that is reported as part of the sleep record.
